# A Soft Sensor Model of Sintering Process Quality Index Based on Multi-Source Data Fusion

**DOI:** 10.3390/s23104954

**Published:** 2023-05-21

**Authors:** Yuxuan Li, Weihao Jiang, Zhihui Shi, Chunjie Yang

**Affiliations:** 1 Hikvision Research Institute, Hangzhou 310051, China; yuxuanli@zju.edu.cn (Y.L.);; 2 State Key Laboratory of Industrial Control Technology, College of Control Science and Engineering, Zhejiang University, Hangzhou 310027, China

**Keywords:** multi-source data fusion, sintering quality prediction, image feature extraction, keyframe extraction

## Abstract

In complex industrial processes such as sintering, key quality variables are difficult to measure online and it takes a long time to obtain quality variables through offline testing. Moreover, due to the limitations of testing frequency, quality variable data are too scarce. To solve this problem, this paper proposes a sintering quality prediction model based on multi-source data fusion and introduces video data collected by industrial cameras. Firstly, video information of the end of the sintering machine is obtained via the keyframe extraction method based on the feature height. Secondly, using the shallow layer feature construction method based on sinter stratification and the deep layer feature extraction method based on ResNet, the feature information of the image is extracted at multi-scale of the deep layer and the shallow layer. Then, combining industrial time series data, a sintering quality soft sensor model based on multi-source data fusion is proposed, which makes full use of multi-source data from various sources. The experimental results show that the method effectively improves the accuracy of the sinter quality prediction model.

## 1. Introduction

In the process industry such as the sintering process, process monitoring is mainly based on conventional sensors which measure the temperature, pressure, flow and other data of the process. The measurement of quality indicators is generally offline testing and it is difficult to achieve online analysis. At present, some online sensors that meet the needs have been developed and applied to some processes with relatively simple reaction mechanisms.

The most important quality index of the sinter is the FeO content of the sinter, which can reflect the reducibility and strength of the sinter. The reducibility of iron ore raw material is an important index for the later blast furnace ironmaking, which determines the furnace conditions and the adjustment of various parameters.

With the increase in FeO content, the output of molten iron in the blast furnace will decrease. However, we cannot simply pursue low FeO content, because FeO content also determines the strength of the sinter. The low content will reduce the strength of the sinter, damage the morphology and increase the proportion of powder. This will hinder the rise of gas flow in the furnace body of the blast furnace, affect the permeability of the material column, make it difficult for the furnace to run smoothly and reduce the output [1].

The common detection method is offline laboratory tests. The operator takes the sintered sample on the conveyor belt and sends it to the chemical composition analysis room. Potassium dichromate titration is often used for offline laboratories and the operation is cumbersome. This method necessitates grinding the sample. If the sample is difficult to dissolve, the analysis results will be poor. However, in general, the method has high accuracy and is the common chemical analysis method for FeO of sinter in iron and steel enterprises [2]. Another test method is X-ray diffraction, which is still an offline detection method. The ore sample to be measured and the supporting internal standard reagents need to be dried in an oven at 105 ${}^{\circ }$C for 2 h and cooled to room temperature before they can be taken. The powder must then be carefully ground to a uniform and consistent level; otherwise, errors may be introduced [3].

The above offline tests are relatively accurate; however, they are time-consuming, often taking three or four hours to produce a result, and the labor and material costs of the tests are high. Whereas the process of sintering ore from batching to completion of sintering takes about one hour, the test results obtained after three or four hours have a significant lag. If the test results do not meet the process requirements, only the current working condition parameter settings can be adjusted to compensate. This is a great challenge for line operators who want to obtain the control objective of consistently good sinter quality.

Therefore, it is urgent to develop online measurement means of sintering quality indicators. Moreover, sophisticated online measurement means are the basis for building an automatic control system for sinter production. At present, online measurement means mainly include magnetic analyzers and online measurement devices. Shougang Group introduced the magnetic analyzer of the Belgium company to detect via cutting the magnetic induction line when the iron ore passes and causing the change of magnetic field current [4]. However, this method has strict requirements for samples and it also needs regular calibration and calibration by the instrument side. Shougang Group reported that the measurement accuracy of the magnetic induction coil was poor because the particles of the measured sinter samples were too small and there were many powder particles and the problem could not be solved by the equipment manufacturer’s personnel again.

As more and more industrial data can be recorded with the use of automated systems in factories, data-driven soft measurement methods are emerging as viable solutions for industrial measurements. Li et al. used LSTM neural networks with self-encoders to construct a prediction model for FeO content [5,6]. Yan et al. proposed a denoised self-encoder framework for predicting sintering endpoints [7]. Yang et al. used a hidden variable model to model sinter quality index [8].

With the development of industrial cameras and image algorithms in recent years, image-based online soft sensors in the field of industrial inspection have gradually gained the attention of academia and industry [9]. Usantiaga proposed a temperature measurement system for the sinter cooling process based on infrared thermal imaging technology [10]. Jiang combined the mechanism with the image characteristics acquired via the infrared imager and proposed a method for measuring the polymorph of FeO content in sinter based on the heterogeneous characteristics of infrared thermal images [11]. However, this method uses the fuzzy classification labels obtained via mechanism analysis and the label accuracy required via regression analysis is insufficient. In addition, the above study used BP neural networks as regressors, which made it difficult to obtain time-series information. And the shallow feature extractor used in this study, cannot obtain deep feature information of the image. In recent years, deep learning methods have been increasingly applied in the image field, such as deep convolutional network models such as ResNet [12,13], achieving better results than shallow networks. This is also one of the motivations for the research in this paper.

This paper combines the low-resolution images of a thermal imaging camera with the high-resolution images of an industrial camera to achieve more accurate access to critical information. Because there are too few assay labels, it is difficult to detect in real time, and more quality variable labels need to be predicted. The work in this paper relies on experts to calibrate a batch of label data and obtains a better database. On this basis, a sintering quality prediction model based on multi-source data fusion is proposed and video data collected via industrial cameras and thermal imagers are introduced. Firstly, the keyframe information of the tail video of the sintering machine is obtained via the keyframe extraction method based on the feature height. Secondly, using the shallow layer feature construction method based on sinter layering and the deep layer feature extraction method based on ResNet, the feature information of the image is extracted from both the deep layer and the shallow layer. Then, the industrial time series data information is fused and the sintering quality prediction model based on multi-source data fusion is designed, which fully extracts the heterogeneous data information from multiple sources. Finally, the method is applied in a practical industrial case. The experimental results show that the model can effectively improve the accuracy of the sinter quality prediction model.

The main contributions of this paper are as follows:

1. Obtaining shallow features based on industrial mechanics with thermal and visible images.
2. Extracting deep features based on the ResNet model and constructing a multi-scale feature extraction model.
3. Fusing time series data to construct a novel soft measurement model based on multi-source data fusion for FeO content of sintered ore.

The remainder of this paper is organized as follows. In Section 2, the characteristics of the sintering process and quality variables are analyzed. The method and model proposed in this paper are introduced in Section 3. Then, the proposed method is verified via the actual production process data in Section 4. Section 5 summarizes the full text and puts forward the new prospect and future work direction.

## 2. Characteristics of Multi-Source Data in Sintering

### 2.1. Description of the Sintering Process and Test Data

**Figure 1:**
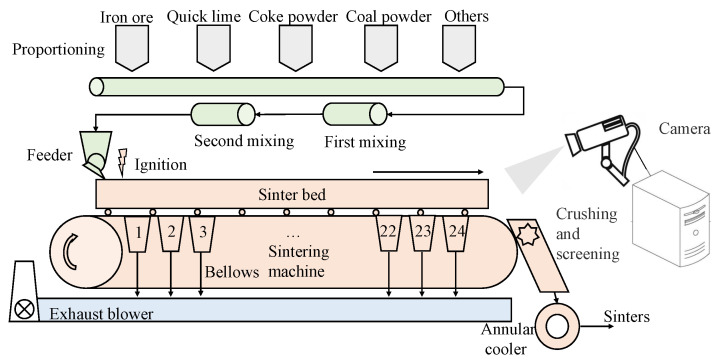
Schematic diagram of image acquisition system of sintering process.

A sintering process is shown in Figure 1. Before the sintered ore is fed into the blast furnace, it is divided into several processes: proportioning, mixing, sintering, crushing, screening and cooling. There are many different types of raw materials used for sinter production. There are more than 10 different bins, consisting mainly of iron ore fines, fuels, fluxes and some additional raw materials. A reasonable material ratio should be developed based on the different compositions of the raw materials, the quality requirements of the sinter ore and the quality requirements of the blast furnace ironmaking. The sintering ore mixing process requires full mixing of the components to obtain a mixture with a uniform and stable chemical composition, while adding water to obtain a good granularity and the necessary material temperature and to improve permeability. The mixture is fed into the belt sintering machine for production and after the sintering is completed, crushing, screening and cooling are required. The finished sintered ore obtained is sent to the ironmaking plant as raw material for blast furnace ironmaking. The sintering data collected contain operational variables, condition variables and quality variables. The quality variables depend on manual testing.

### 2.2. Sintering Image Data Acquisition

**Figure 2:**
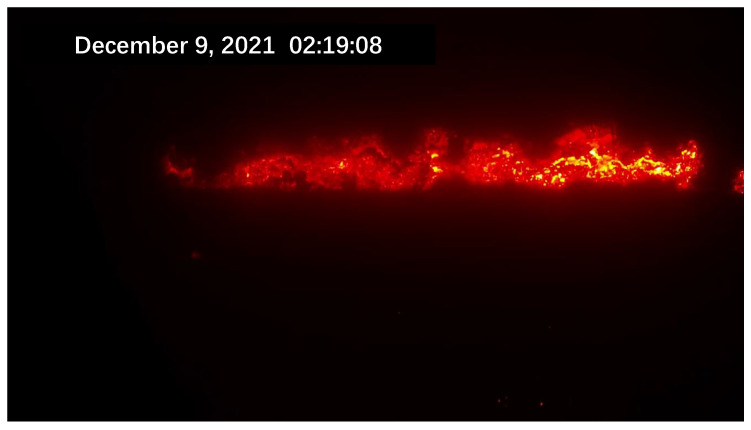
The visible light image acquired via the sintering system.

**Figure 3:**
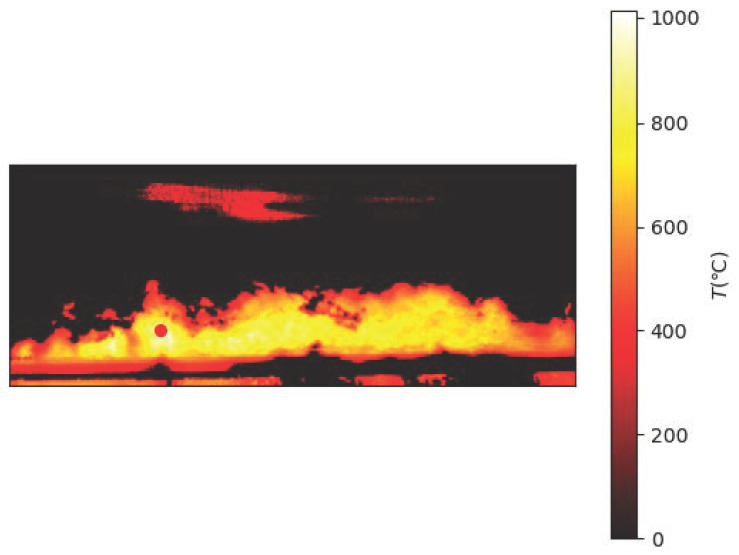
Thermal image acquired via the sintering system.

Due to the scarcity of laboratory data, the sintering process information is not perfect. To better obtain information on the sintering process, an industrial camera and a thermal camera were set up at the observation port at the end of the sintering machine. A schematic diagram of the sintering machine image acquisition is shown in Figure 1. The industrial visible light camera has a resolution of 1920 × 1080 and the captured images are shown in Figure 2. The thermal imager has a resolution of 640 × 480 and the captured image is shown in Figure 3.

The captured information is uploaded to the database for storage via the industrial fieldbus. Live video information is displayed on a display screen in the central control room for the controller to view or can be downloaded to a cloud server via a remote connection.

### 2.3. Analysis of Sintering Image Data

At present, most of the quality indexes of sinter are obtained via laboratory analysis. In the process of sinter production, due to the lag of laboratory analysis, the actual working conditions often depend on expert experience to judge. During the sintering process, the internal state of the sinter is not visible and only the topmost surface is exposed. However, the topmost part is the part that is ignited and sintered first. Therefore, on the sintering machine with a length of 90 m, most of the length is in a sintered and finished state. Only the flatness information about the ore laying can be observed from the top and the key sintering state information cannot be obtained. Fortunately, once sintering is complete, the sintered ore reaches the end of the trolley and falls naturally into the collection bin. After the last piece of sintered ore has fallen and before the next piece of sintered ore falls, we can observe the flame information in the sinter section. There is a flame viewing port at the end of the sintering machine. Experts with rich production experience can analyze the range of the FeO quality index of the sinter at the moment by observing the flame red layer data at the end of the sintering machine. Through such manual judgment, it is possible to rely on expert experience to help judge and control the process in the absence of sufficient test indicators. However, there are still significant limitations to this method, with the subjective errors of manual judgement being high and subject to the harsh conditions of the industrial site. In harsh conditions such as high temperatures, loud noises, vibrations and dust, it is difficult for workers to carry out observations for long periods of time, making it difficult to replace conventional observation with fire observation as an aid. Therefore, in the actual production process, it is necessary to develop stable measurement means to replace expert manual observation. Experts can make judgements by observing flame images. Therefore, in this experiment a camera is set up to capture the images and learn information and it is feasible to gauge experts’ levels of experience and knowledge through soft measurements.

Due to the different sintering states of raw materials in different directions and different temperatures, the flame colors presented by them are also different. The surface of the black part is sintered and the temperature is reduced to below 300 ${}^{\circ }$C, resulting in dim color and unclear observation. The red fire layer is the burning part of the sinter. It is generally located in the lower part of the sinter, mainly in red, and the temperature is about 600 ${}^{\circ }$C. The stomatal layer is the brightest part of the image. Because the temperature exceeds 800 ${}^{\circ }$C, it appears yellow and white in the image. The sinter will fall periodically at the tail of the sintering machine. When a batch of sintered ore reaches the last wind box at the end of the sintering machine, it will break and fall with the rest of the ore still on the trolley due to the loss of the support of the trolley. At the moment of falling, the flame information of the fault is very clear. After a short time, the falling of ore will raise a large amount of dust and cause vibration at the same time, making the image captured via the camera blurred and accompanied by shaking. It is difficult to obtain accurate flame information from the image at this time. Therefore, the system needs to screen out the clear images at the moment of falling as the analysis sample set. Experienced workers can judge the FeO content of the sinter at this time by observing the section.

## 3. The Soft Sensor Model Based on Multi-Source Data Fusion

### 3.1. Keyframe Extraction from Video Image of Sintering Machine Tail

In this experiment, the monitoring video is obtained from the sintering monitoring system for preprocessing. Since the original color image is three-channel RGB data with a data dimension of 16.77 million, the processing is relatively complicated and the calculation pressure is relatively large, so it is grayed first. The grayscale calculation formula is as follows:

(1) $$\begin{array}{c} Gray=Blue\times 0.114+Green\times 0.587+Red\times 0.299 \end{array}$$

where *red*, *green* and *blue*, respectively, represent the values of red, green and blue channels and gray is the gray value.

**Figure 4:**
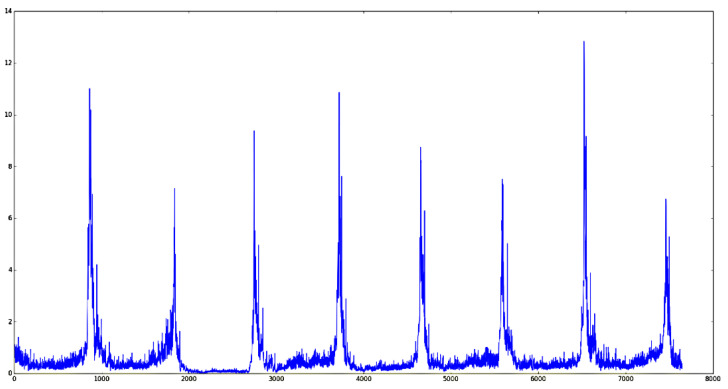
Graph of raw inter-frame difference intensity.

**Figure 5:**
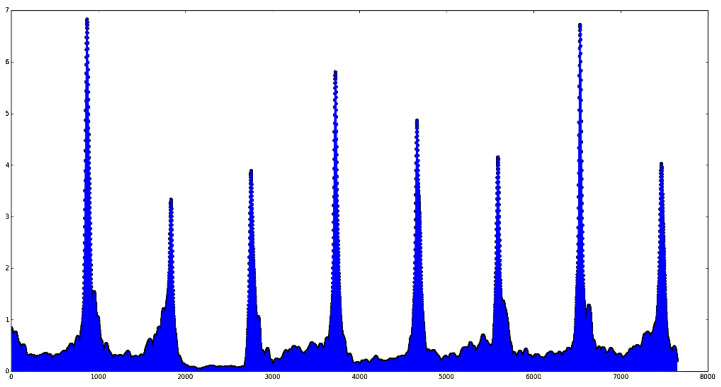
Graph of smmoothed inter-frame difference intensity.

According to the monitoring video, the falling of sinter is a periodic process. When a batch of ore arrives at the tail, the front part breaks and falls, exposing the red fire layer of the section. The image at this time is the keyframe required for the experiment. After that, this batch of ore will continue to fall and its shape will change in the air and at the same time it will raise smoke and dust, which is unfavorable for observing its sintering characteristics. Therefore, we need to obtain these keyframes in the video as our input. The inter-frame difference method is often used for the algorithm of acquiring the keyframe of the target motion when video monitoring the moving target [14]. The algorithm performs differential processing on continuous images in continuous time. The difference in the grayscale is calculated by subtracting the pixel values of different frames. Then, the threshold value of the gray difference is set. When the value exceeds the threshold value, this frame is selected as the keyframe, indicating that the monitoring target has changed greatly from the previous time at this frame time. When the inter-frame difference method is used to monitor the red layer at the tail of the sintering machine, the brightness difference between different frames can be used as the threshold control to select the keyframe. Since the falling of sinter is a periodic process, when the current ore falls to the bottom and the new batch of ore has not yet broken down, the brightness of the image is the lowest. After a new batch of ore appears, the image information will increase rapidly. Through this characteristic, the sinter keyframe extracted based on the inter-frame difference intensity is the required cross-sectional image. For each frame, the difference is made with the previous frame to obtain the different strengths between the frames and the drawing is as shown in Figure 4. After smoothing, each extreme point can be extracted as our keyframe and the result is shown in Figure 5.

**Figure 6:**
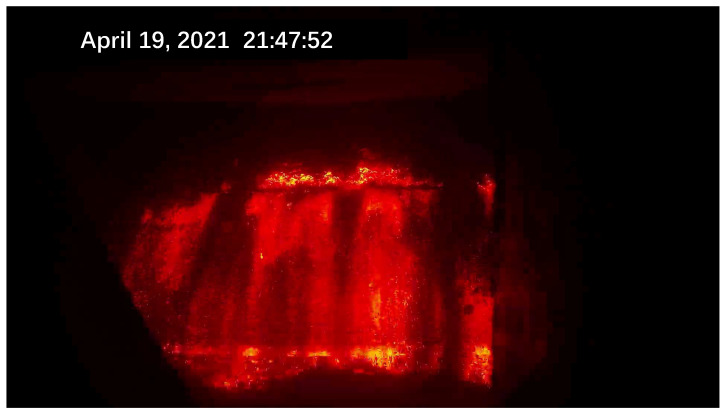
Abnormal frames extracted via the inter-frame difference algorithm.

However, the accuracy of the inter-frame differencing algorithm depends on the choice of threshold values. In addition, there are some abnormal states during the falling process of the sinter. The sinter disintegrates in the air due to collision and other reasons, resulting in sudden changes in image intensity. As shown in Figure 6, abnormal frames are extracted and need to be manually removed during calculation.

Because of the limitation of the algorithm based on inter-frame difference, a more intuitive keyframe extraction algorithm based on feature height is proposed in this experiment.

**Figure 7:**
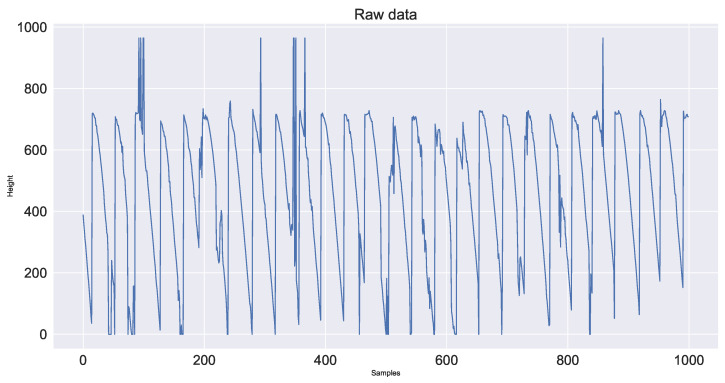
Plot of feature height.

**Figure 8:**
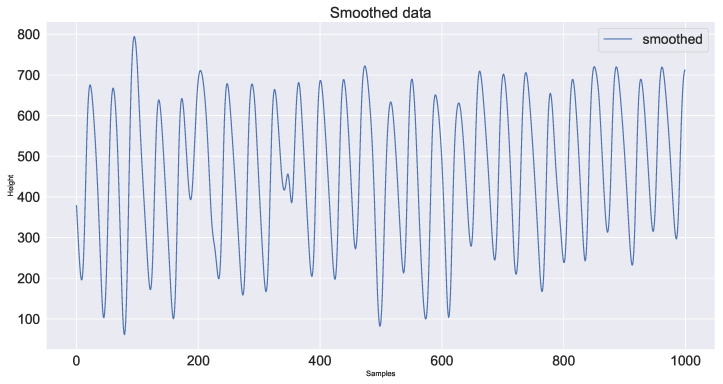
Plot of smoothing feature height.

First, mask segmentation based on the gray threshold is performed on the image that has been converted to gray level by mask and the segmented image is the red fire layer area to be monitored. Next, the contour of the red fire layer is obtained by using the findcontours algorithm. According to the contour, the circumscribed rectangle is fitted to represent the current sintering red layer. The center of gravity of this rectangle is the feature height. The feature height of the video collected in this experiment is shown in Figure 7 and the periodic feature height fluctuation diagram obtained after smoothing is shown in Figure 8.

**Figure 9:**
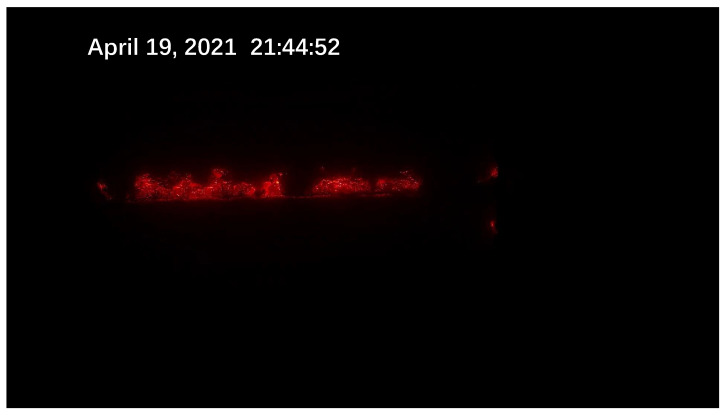
Keyframes extracted via the characteristic height algorithm.

Because the sinter falls periodically, the characteristic height of the red layer of the sinter section is at the highest point when the red fire layer we need has just appeared. Therefore, according to the peaks extremum algorithm, the maximum value of the feature height is obtained and its abscissa is the index of the required keyframe, that is, the image keyframes when the red fire layer has just appeared. The keyframe is extracted via the algorithm based on the characteristic height of the red layer of the sinter and then saved, as shown in Figure 9. The inter-frame intensity difference method of keyframe extraction is disturbed by anomalous image intensity variations, as shown in Figure 6. The feature height method, designed in this paper, is a combination of specific processes where the intensity changes abruptly, but the feature height does not change abruptly, so keyframes are extracted more accurately.

### 3.2. Construction and Extraction of Image Shallow Features

Using the video keyframe extraction algorithm for the red layer at the end of the sinter in the previous section, several image keyframes were obtained for this experiment. For each keyframe image, the sintered red layer appears at the same position at the top. This location is our region of interest (ROI). First, we fix the region and calculate the area of the region as a fixed observation window. Again, the keyframe is segmented by using a mask based on a grey threshold. Based on practical industrial experience and mechanisms, sintered ore has different sintering states and corresponding temperatures. Assisted by an infrared thermal imager for temperature measurement, the layered temperature is converted into an image threshold. Four different thresholds are chosen, as shown in Table 1.

**Figure 10:**
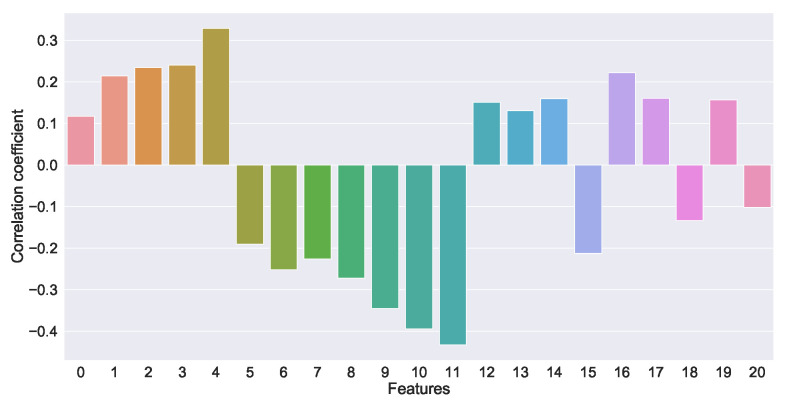
Correlation between shallow features of sintering machine tail image and time series data features.

According to the threshold segmentation, four regions are obtained and then their contours are obtained respectively and the area around each contour is calculated as a feature. To better extract the feature information of the image, the area ratio of the area surrounded by each contour to the ROI is further calculated. The proportion information represents the information of the red fire layer and reflects the distribution characteristics and temperature characteristics of the sintering fault, to calculate the shallow layer characteristic information of the ROI area. The shallow layer features and time series data features of the red layer image at the tail of the sintering machine are extracted and the correlation is shown in Figure 10. Features 0, 1, 2 and 3 in the figure are shallow features, while the remaining features are features of industrial time series data. The shallow features are concatenated with the time series data features for fusion.

### 3.3. Deep Feature Extraction Based on ResNet

**Figure 11:**
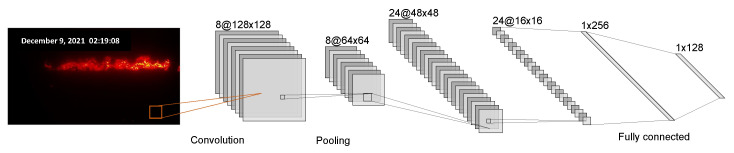
Schematic diagram of CNN structure for identifying numbers.

Convolutional neural network (CNN) is a neural network modeled on human visual mechanisms. When humans first saw an image, they could not judge the whole image. Instead, different nerve cell receptive fields transformed the image into different features for recognition. Similar to the receptive field in human visual cortex cells, CNN extracts the features of images through convolution kernels. The convolution kernel successively crosses the picture with the set convolution step in order and calculates the convolution results of the elements of the picture in the receptive field. As a computer, the ability to recognize images is not as good as human beings. After an image is simply rotated, the matrix information stored in the computer is completely different. Therefore, the convolution kernel is designed to extract features for computer image recognition by imitating the human visual perception field. After convolution, multi-level features such as color and contour can be extracted from the original image to help identify the image [15]. For images that cannot be recognized via single-layer convolution, multi-layer convolution needs to be stacked to extract deep features. However, after convolution, the dimension of the original image will be increased. Simply stacking convolution layers will cause the dimension to be too high and it is difficult to train the depth network. Therefore, it is necessary to perform a pooling operation after convolution. The structure is shown in Figure 11. By calculating the mean value or the maximum value of the rectangular window of the specified size to replace the original rectangle, dimension reduction can be completed and the function of nonlinear mapping can be achieved. The stacking of convolution and pooling layers has a good effect within 20 layers. However, after increasing the number of layers of the network, the performance of the model does not improve, but will decline. when the network depth is increased, the network performance will neither improve nor even deteriorate [16].

**Figure 12:**
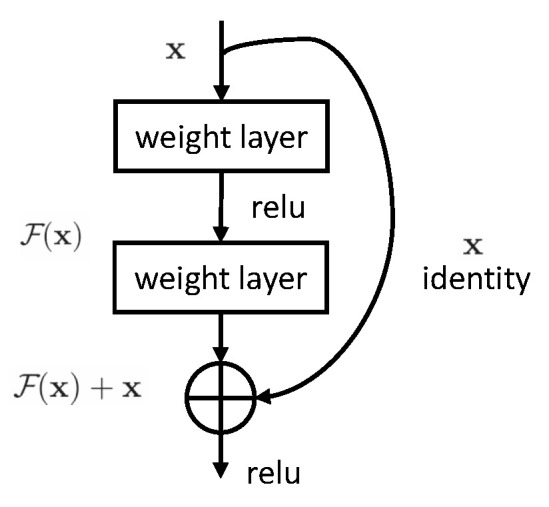
Schematic diagram of residual connection structure.

To solve this problem, He et al. proposed a deep residual network (ResNet), which sets a residual connection between every two layers [12]. Similar to the circuit short-circuit mechanism, the residual connection method constructs an identity map, which enables the network to have the ability of layer hopping transmission. When the number of stacked layers is too deep, the weight of training can select layer hopping connection, as shown in Figure 12. The original function mapping is expressed as H(x). Here, we build another map F(x)=H(x)−x that is used to fit stacked networks. At this time, the original mapping is transformed into H(x)=F(x)+x, which can be achieved by a short circuit connection. Such processing does not increase the number of additional parameters, nor does it increase the computational complexity, and it is still easy to train.

The network achieved good results in Imagenet image recognition. For the deep stacked network, the residual connection block set between every two layers can be expressed as:

(2) $$y=F(x,\;\left\{W_{i}\right\})+x.$$

x, y are the input vector and output vector of the residual layer, respectively. $F(x,\;\left\{W_{i}\right\})$ is a representation of the residual map that needs to be learned.

**Figure 13:**
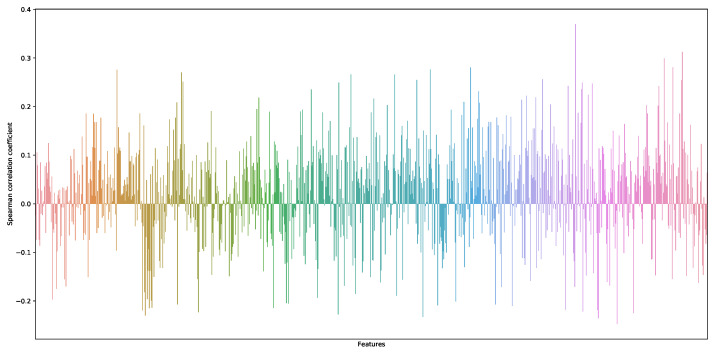
Schematic diagram of deep feature correlation extracted via ResNet model.

**Figure 14:**
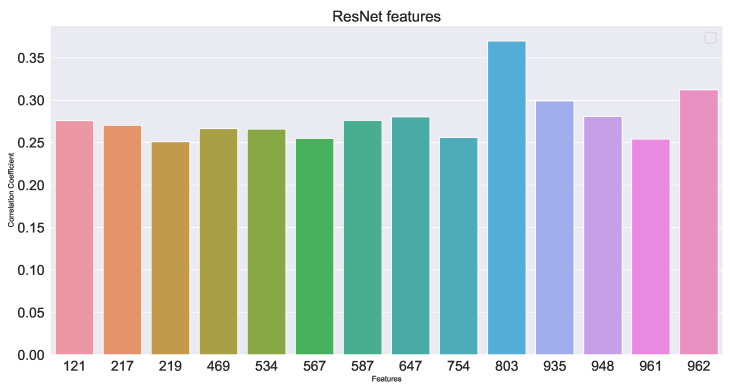
Schematic diagram of selected deep feature correlation.

The trained network classifier ResNet-50 is used in this experiment and the 1000 output of its classification is used as the deep feature fixed extractor of the experiment. A total of 1000 deep-seated features were obtained through the ResNet model and their correlation was analyzed, as shown in Figure 13. The features were selected with the correlation coefficient greater than 0.25 as the deep features of the red layer image at the tail of the sintering machine, as shown in Figure 14.

### 3.4. Prediction Method of Sintering Multi-Source Data Fusion

**Figure 15:**
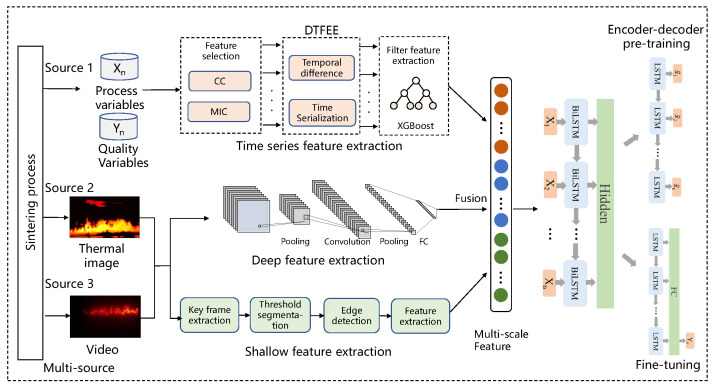
Diagram of Sintering prediction model based on multi-source data fusion.

Because the data information from many different sources can not be directly extracted and utilized, we propose a prediction method of the sintering process based on multi-source data fusion. The shallow features obtained via keyframe extraction, threshold segmentation, edge detection and feature construction and the deep features obtained via ResNet model are connected to obtain our deep and shallow fused image features. The time series data information from the process is processed using the encoder–decoder dynamic time features expanding and extracting method (DTFEE) based on the LSTM network [5,6]. The characteristics of the image, together with the characteristics obtained from the time series data information of the process, are used as the input of the encoder–decoder model to train the predicted output of the quality variable. The block diagram of the multi-source data fusion prediction method combined with deep and shallow image features is shown in Figure 15.

## 4. Case Study on Sintering Process

### 4.1. Introduction to the Multi-Source Dataset of Sintering Process and Settings

The images used for the soft sensor of the sinter are collected via the thermal imager and industrial camera, respectively. Video data were collected from 8 December 2021 to 10 December 2021. The acquisition resolution of the industrial camera is 1920 × 1080 and that of the thermal imager is 640 × 480. Using the feature height method, the keyframes are obtained from the video for expert calibration. The extracted deep and shallow features and process time series data features jointly construct a dataset, with a total of 1319 samples. Each sample has 35 features, including 17 time-series features, 4 shallow features and 14 deep features. There are 1100 sets of samples in the training set and 219 sets of samples in the test set. The model consists of two layers of LSTM, with 50 hidden layer units and an input length of 50. The optimizer uses Adam and the early stop step is set to 20.

### 4.2. Comparison of Results and Analysis

**Figure 16:**
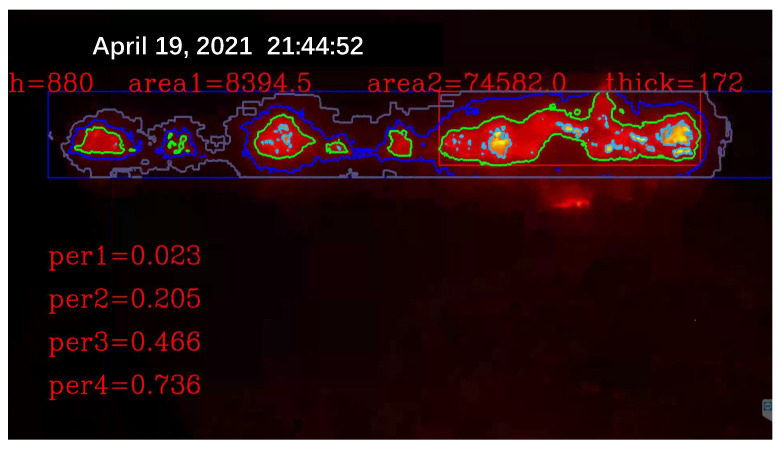
Video feature extraction diagram of the red layer at the end of the sintering machine.

The effect of shallow feature extraction is shown in Figure 16. The online acquisition of characteristic height, the thickness of the red flame layer, the distribution area and the proportion of four flame layers are presented.

In this experiment, the accuracy of the prediction model was evaluated by mean square error (MSE), mean absolute error (MAE) and hit rate (HR), where *y* and $\hat{y}$ are the real value and prediction, respectively, and *n* is the number of test samples. See Table 2 for the average evaluation indexes of the model obtained from the 10 experiments.

(3) $$\begin{array}{cc} MSE & =\frac{1}{n}\sum_{i=1}^{n}{\left(y_{i}-{\hat{y}}_{i}\right)}^{2} \end{array}$$

(4) $$\begin{array}{cc} MAE & =\frac{1}{n}\sum_{i=1}^{n}\left|y_{i}-{\hat{y}}_{i}\right| \end{array}$$

(5) $$\begin{array}{c} \begin{array}{c} H_{i}=\left\{\begin{array}{cc} 1, & \left|y_{i}-{\hat{y}}_{i}\right|/y_{i}<=1.5\%\\ 0, & \left|y_{i}-{\hat{y}}_{i}\right|/y_{i}>1.5\% \end{array}\right. \end{array} \end{array}$$

(6) $$\begin{array}{cc} HR & =\frac{1}{n}\sum_{i=1}^{n}H_{i} \end{array}$$

**Figure 17:**
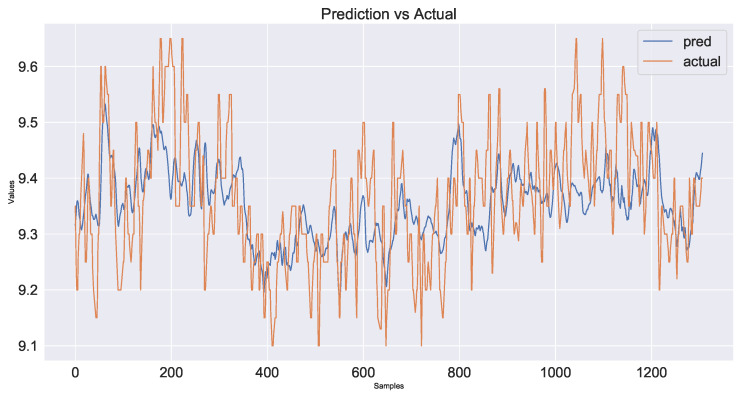
Plot of prediction results using time series data for industrial processes only.

**Figure 18:**
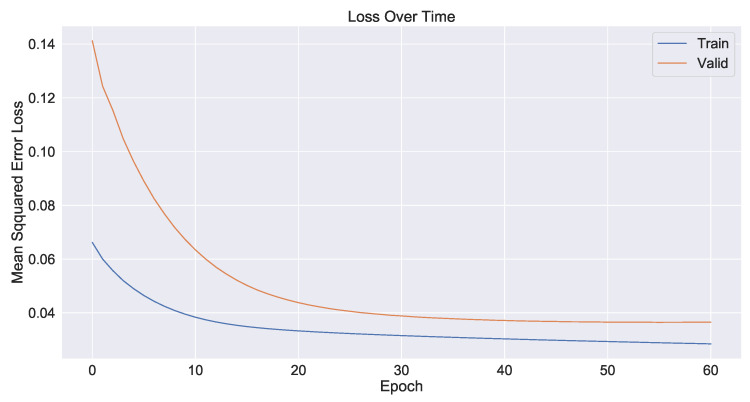
Plot of the loss function of the time series data model during network training.

**Figure 19:**
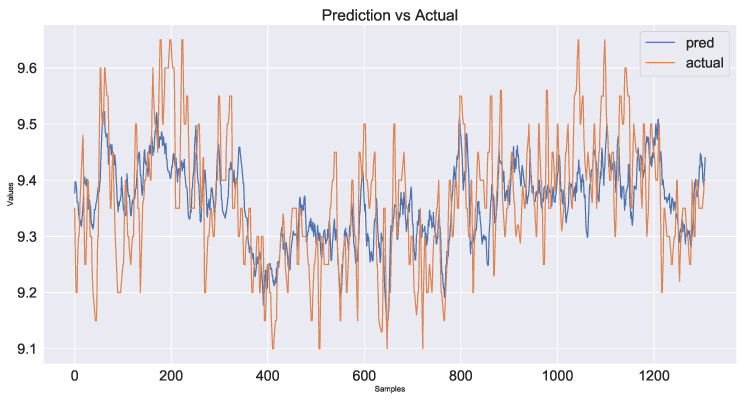
Plot of prediction results using time series data and shallow feature fusion model for industrial processes.

**Figure 20:**
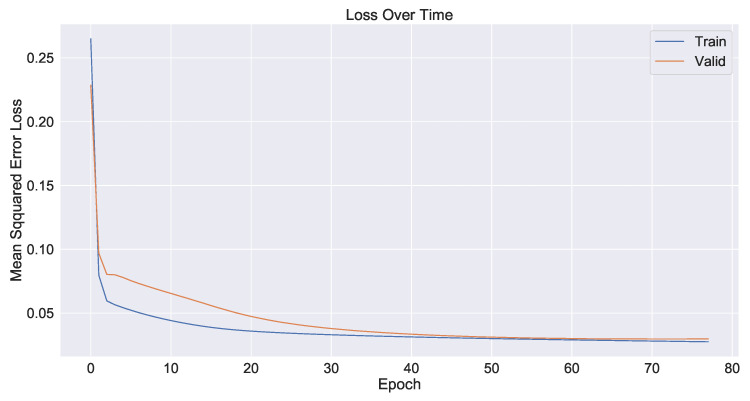
Plot of the loss function of time series data and shallow feature fusion model during network training.

**Figure 21:**
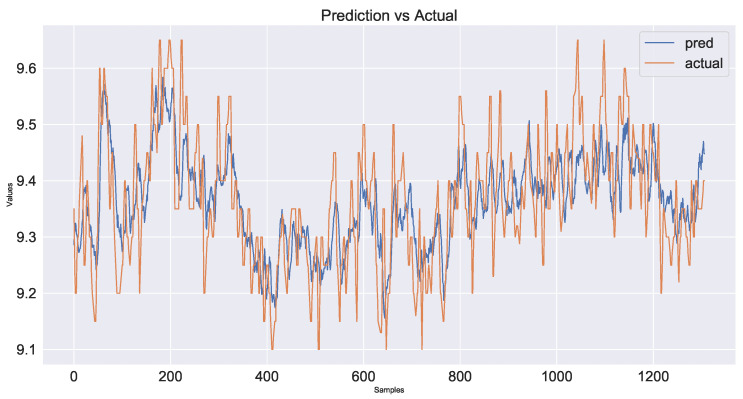
Plot of prediction results using time series data, shallow feature and deep feature fusion model for industrial processes.

**Figure 22:**
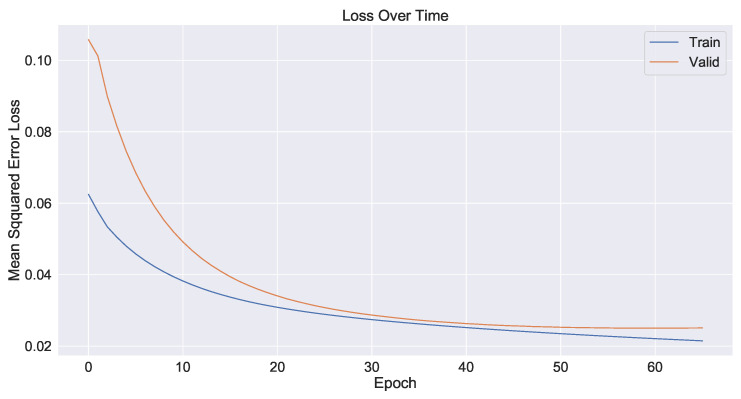
Plot of the loss function of time series data, shallow feature and deep feature fusion model during network training.

As a control group, the model used only time series data from the industrial process and did not use image information fusion. The prediction results are shown in Figure 17 and the decline in the loss function during network training is shown in Figure 18. The second model added shallow image information fused with time series data. The prediction results are shown in Figure 19 and the loss function decline diagram during network training is shown in Figure 20. The prediction results of the model fused with the deep and shallow image information are shown in Figure 21 and the decline in the loss function when training the network is shown in Figure 22.

It can be seen from Table 2 that the prediction model of multi-source data fusion proposed in this paper achieved good results and fast convergence speed. Compared with the method using only time series data and shallow features, the model fusing time series data, deep and shallow features obtains more process information and rich features at different scales, and the prediction effect is improved to some extent.

Figure 10 and Figure 14 show that the correlation of the extracted deep and shallow features is similar to that of the time series data, which has some significance for the prediction model.

Compared with the industrial process time series data model without introducing images, the MSE of the deep and shallow image information model proposed in this paper decreases by about 29% and the hit rate increases from 86.5% to 93.1%. Compared with the temporal data fusion shallow feature model, the MSE of the fusion deep and shallow image information model proposed in this paper decreases by about 24% and the hit rate increases from 89.8% to 93.1%. The actual industrial application verifies the effectiveness of the multi-source data fusion method proposed in this paper.

## 5. Conclusions

This paper presents a method to detect FeO content in sinter based on multi-source information fusion. The method first collects video data of the red layer at the end of the sintering machine through an industrial camera. Secondly, the keyframe extraction algorithm based on feature height and the shallow feature construction method based on sinter layering are designed according to the actual process. Then, deep features of sinter tail red layer images are extracted from keyframes by ResNet model. Finally, combined with the process parameters of the production process, an online real-time prediction model of FeO content in sinter is established through the LSTM network.

The model solves the problems of poor time efficiency and high cost of existing technologies by extracting multi-scale information from industrial camera video data and integrating the process parameters. It has practical significance for the guidance of the sinter production process and provides technical support for energy conservation, emission reduction, and quality and efficiency improvement of iron and steel enterprises. There are also quality variables in the sintering process that are relevant to the images of the faults, such as the total iron content, tumbler index, etc. This method can be extended to other variable predictions as long as suitable image labeled data are available. However, the new system requires labeled data before deployment, high quality image labeled data have a direct impact on system accuracy. To reduce manual effort and improve deployment efficiency, a future direction that could be considered is self-supervised learning of images, thus reducing the workload of expert labeling.

## Figures and Tables

**Table 1:** Image threshold selection table.

Threshold	Meaning	Temperature (${}^{\circ }$C)
100	Gas hole layer	800
40	Red fire layer	600
20	Dark fire layer	400
10	Sintering finished layer	200

**Table 2:** Evaluation index of prediction value of different methods.

Methods	MSE	MAE	HR
Time series data	0.0082	0.072	0.865
Time series data + shallow feature fusion	0.0076	0.069	0.898
Time series data + Deep and shallow feature fusion (ours)	0.0058	0.061	0.931

## Data Availability

Not applicable.
